# Association between noise exposure during pregnancy and pregnancy complications: A meta-analysis

**DOI:** 10.3389/fpsyg.2022.1026996

**Published:** 2022-11-21

**Authors:** Zihao Wang, Rongkai Qian, Wanwan Xiang, Landi Sun, Mengmeng Xu, Boxing Zhang, Liren Yang, Sijing Zhu, Lingxia Zeng, Wenfang Yang

**Affiliations:** ^1^Department of Obstetrics and Gynecology, Maternal and Child Health Center, The First Affiliated Hospital of Xi'an Jiaotong University, Xi'an, China; ^2^The First Affiliated Hospital, Xi'an Jiaotong University Health Science Center, Xi'an, China; ^3^School of Public Health, Xi'an Jiaotong University Health Science Center, Xi'an, China

**Keywords:** noise exposure during pregnancy, noise pollution, pregnant women, pregnancy complications, hypertensive disorders of pregnancy (HDP), gestational diabetes mellitus (GDM)

## Abstract

**Background:**

Noise exposure has a significant impact on human health. However, the effect of occupational and residential noise on the risk of pregnancy complications was controversial in the literature. This study looked at previous research and performed a meta-analysis to determine how noise exposure during pregnancy affected the risk of pregnancy complications.

**Methods:**

Systematic searches were conducted in PubMed, Web of Science, Scopus, Embase, Ovid, and Cochrane, and all relevant studies were included. Two investigators independently evaluated the eligibility of these studies. The risk of bias in each study and the quality and strength of each outcome was evaluated by using the GRADE approach and Navigation Guide. Random effects meta-analysis model was used.

**Results:**

The meta-analysis retrieved 1,461 study records and finally included 11 studies. Occupational noise exposure during pregnancy was associated with preeclampsia (*RR* = 1.07, 95%*CI*: 1.04, 1.10). Neither occupational nor residential noise exposure was associated with hypertensive disorders of pregnancy (HDP) (*RR* = 1.10, 95%*CI*: 0.96, 1.25 and *RR* = 1.05, 95%*CI*: 0.98, 1.11) or gestational diabetes mellitus (GDM) (*RR* = 0.94, 95%*CI*: 0.88, 1.00 and *RR* = 1.06, 95%*CI*: 0.98, 1.16). Further bias analysis showed that the results were reliable. All outcomes were rated as low in quality and inadequate evidence of harmfulness in strength.

**Conclusions:**

Occupational noise exposure could increase the risk of preeclampsia, according to the findings. There was no clear evidence of a harmful effect of noise exposure during pregnancy on HDP or GDM.

## Introduction

Noise, or unwanted sound, mainly consists of community and workplace noise (Min and Min, 2017). As urbanization and industrialization continue to expand, noise pollution continues to increase (Lee, 2002; Teixeira et al., 2021a). Among environmental factors affecting people's health, traffic noise ranks second only to particulate air pollution. It was estimated that traffic noise pollution costed western European nations at least 2 percent of their annual gross domestic product and one million disability-adjusted life years (WHO, 1997, 2011; Hanninen et al., 2014; Teixeira et al., 2019; Themann and Masterson, 2019). Additionally, occupational noise has been linked to a number of negative health outcomes, including hearing loss and cardiovascular disease (WHO, 2004; Nieuwenhuijsen et al., 2017).

Noise is a type of environmental stressor that can cause excessive activation of the automatic nervous system (Golmohammadi et al., 2022), long-term activation of the autonomic nervous system can lead to its dysfunction, resulting in hypertension, gastrointestinal dysfunction, sleep disorders, etc (Hume et al., 2012; van Kempen and Babisch, 2012; Roswall et al., 2018). Noise above 85 decibels can directly lead to irreversible hearing impairment. In addition, noise is considered a risk factor for a number of chronic diseases due to its mental effects, which include anxiety, irritability, and high stress levels (Munzel et al., 2014; Dzhambov, 2015; Golmohammadi et al., 2022). Noise can also affect pregnant women and their fetuses, leading to adverse pregnancy outcomes. Multiple studies and meta-analyses have demonstrated that noise contributes to low birth weight, preterm birth, miscarriage, stillbirth, and congenital malformations (Dzhambov et al., 2014; Patelarou and Kelly, 2014; Pedersen et al., 2017a; Poulsen et al., 2018; Selander et al., 2019; Smith et al., 2020; Yue et al., 2020).

Hypertensive disorders of pregnancy (HDP), including gestational hypertension, preeclampsia, eclampsia and hemolysis, elevated liver enzymes, and low platelets (HELLP) syndrome, and gestational diabetes mellitus (GDM), are among the most prevalent obstetric diseases, affecting between 1 and 15 percent of pregnancies worldwide (Abalos et al., 2013; Chiefari et al., 2017). Preeclampsia and eclampsia are major causes of maternal mortality (Steegers et al., 2010; Say et al., 2014). Moreover, women diagnosed with HDP have an increased risk of developing cardiovascular disease in the future (Steegers et al., 2010). Similarly, both GDM patients and their progeny have an elevated risk of developing type 2 diabetes (Chiefari et al., 2017). The etiology of HDP and GDM is not fully understood, but several risk factors, including aging, obesity, and air pollution, have been repeatedly cited (Sibai et al., 2005; Pedersen et al., 2017b; McIntyre et al., 2019).

Several studies had linked environmental noise exposure to cardiovascular disease and diabetes in the general population (van Kempen and Babisch, 2012; Munzel et al., 2014). However, there was insufficient evidence to draw definitive conclusions regarding the link between noise and pregnancy complications. In Nurminen and Kurppa (1989), published the first study examining the association between occupational noise and HDP among pregnant workers. Since then, several studies had investigated the association between pregnancy complications and residential or occupational noise exposure. Some studies confirmed that noise exposure could lead to an increased prevalence of HDP, while a few studies demonstrated the opposite. Furthermore, opinions regarding the correlation between noise exposure and GDM were quite divergent. Min and Min (2017) evidenced that noise exposure significantly increased the risk of GDM, whereas Thacher et al. (2021) concluded that noise had the opposite effect on GDM. There was no meta-analysis of the relationship between different types of noise exposure and pregnancy complications, with results varying from study to study.

Therefore, we conducted a meta-analysis of cohort and case-control studies to investigate comprehensively the association between various types of noise exposure and the risk of pregnancy complications.

## Methods

### Search strategy

Our review was registered with PROSPERO (www.crd.york.ac.uk/PROSPERO) under protocol number CRD42021245124 and developed in accordance with the PRISMA guidelines (www.prisma-statement.org). We utilized six search engines, including PubMed, Web of Science, Scopus, Embase, Ovid, and Cochrane library, to conduct a systematic search (studies published before September 30, 2022). Two researchers conducted independent searches. We used the terms of “pregnan^*^,” “gestat^*^,” “impregnat^*^,” “cyes^*^,” “hypertensi^*^,” “diabet^*^,” “mellitus^*^,” “hyperglyc^*^,” “eclampsia^*^,” “nois^*^,” “decibel^*^,” “voic^*^,” “sound^*^,” “loud^*^,” “high blood pressure,” “HBP,” “HDP,” “HELLP,” and “GDM.” In addition, we used terms like “epidemi” and “cohort” to limit the types of studies included. The detailed search strategies of each database were shown in Supplementary Appendix A. In addition to the database search, we conducted manual searches for the following types of studies: journal articles pertaining to environmental exposure and maternal and child health, as well as reference lists of the included studies. After deduplication, two researchers independently evaluated the eligibility of studies and downloaded and read their full texts for additional screening (Teixeira et al., 2019, 2021b). Articles were evaluated on three levels: the title, the abstract, and the full text.

### Inclusion and exclusion criteria

Study question: Whether exposure to ambient noise increases the maternal risk of pregnancy complications.

Population: Pregnant women with gestational age ≥20 weeks were included. Children (age <15 years), pregnant women with gestational age <20 weeks, women who had a miscarriage, and women with high blood pressure or diabetes prior to pregnancy were excluded.

Exposure: All studies on the relationship between noise and pregnancy complications were included, whether occupational noise or residential noise. There were objective noise measurements (e.g., model evaluations or field measurements), semi-objective noise measurements (e.g., expert evaluations) and worker self-reports. If a study reported both objective and subjective measures, the objective measure was given precedence.

Comparators: Comparators were considered for participants exposed to the lowest levels of noise. We excluded all other comparators.

Outcomes: This systematic review included five outcomes:

1. Pre-eclampsia superimposed on chronic hypertension (ICD-10 O.11);
2. Gestational [pregnancy-induced] hypertension (ICD-10 O.13);
3. Pre-eclampsia (ICD-10 O14.0, O14.1, O14.2, O14.9);
4. Eclampsia (ICD-10 O15.0, O15.1, O15.2, O15.9);
5. Diabetes mellitus arising in pregnancy (ICD-10 O24.4, O24.9).

Studies with outcomes defined according to the International Classification of Diseases (ICD) were included. In addition, studies that did not use the ICD but which reported outcomes consistent with the ICD were included.

The following measurements were eligible for inclusion:

(i) Diagnosis made by a medical practitioner through medical examination and laboratory tests;
(ii) Hospital discharge records;
(iii) Eligible pregnancy complication registration data;
(iv) Diagnosis confirmed by a doctor after self-report;

Other measures are not permitted.

### Data extraction

After the screening, two researchers (ZW and RQ) independently extracted data from qualified studies using a standard data extraction sheet containing the following information: (1) authors, publication year, and study period; (2) study design; (3) study location; (4) sample size; (5) definition of noise exposure; (6) exposure type; (7) measurement method of noise exposure; (8) diagnostic criteria for HDP or GDM; (9) covariates. Noise exposure was measured using A-weighted decibels [dB(A)] in all studies. In the majority of the included studies, noise levels were analyzed separately for different time periods, and when available, we preferred to use 24-h noise level measurements. In the studies that provided different types of noise (road/rail/aviation), road noise data would be included, because road noise caused more residential noise than rail or aviation noise. Due to the extremely low incidence of eclampsia and to maintain consistency in subgroups between studies, eclampsia and preeclampsia were grouped together as preeclampsia. If there was a disagreement, it should be resolved through group discussion; if disagreements persisted, the third investigator (LY) should intervene and render a decision.

### Bias, quality, and strength assessments

During the data extraction process, the Navigation Guide evaluated the availability of evidence (Johnson et al., 2014). The risk of bias was evaluated for each study included. Regarding the risk of bias tool developed by the Cochrane Collaboration (Lam et al., 2014), each risk of bias was categorized as “low risk,” “probably low risk,” “probably high risk,” “high risk” or “not applicable.” Each study's bias was separated into eight distinct components (Balshem et al., 2011). The researchers evaluated the potential for bias in each of these elements.

We assigned a predetermined initial quality rating (“high,” “moderate,” or “low”) to the body of evidence, in reference to the evidence-based decision making approach used in the clinical sciences to make medical interventions, namely Grading of Recommendations Assessment Development and Evaluation (GRADE) (Guyatt G. et al., 2011; Teixeira et al., 2019). The quality ratings were then modified (downgraded or upgraded) based on the characteristics of each study component (Balshem et al., 2011). As recommended by Navigation Guide, human observational studies were initially rated as “moderate” in terms of quality (Woodruff and Sutton, 2014). The possible ratings were 0 (no change from “moderate” quality), −1 (one-level downgrade), −2 (two-level downgrade); +1 (one-level upgrade) or +2 (two-level upgrade) (Balshem et al., 2011; Guyatt G. H. et al., 2011). We assessed the overall strength of the evidence according to a combination of four criteria: (A) Quality of the body of evidence (i.e., the rating from the previous step); (B) Direction of the effect estimation; (C) Confidence in the effect estimate; and (D) Other compelling attributes of the data that may influence certainty. According to the Navigation Guide, there were four levels of evidence strength: “sufficient evidence of toxicity,” “limited evidence of toxicity,” “inadequate evidence of toxicity,” and “lack of evidence of toxicity.” The evaluators should independently evaluate the strength of the evidence based on four criteria and provide an appropriate description.

### Statistical analysis

The RevMan software (Review Manager [Computer program]. Version 5.4.1, The Cochrane Collaboration, 2020) provided by the Cochrane Collaboration was used for meta-analysis. Relative risk (*RR*) was used as the analysis statistic of influence. Due to the low prevalence of the pregnancy complication we studied, the difference between odds ratios (*OR*) and *RR* was relatively small, and we interpreted all reported statistics as *RR*. Each effect was expressed by a 95% confidence interval (95%*CI*). Dose-response conversion was performed using the Generalized least squares method developed by Orsini et al. (2006). χ^2^ test was used to analyze the heterogeneity among the studies, and *I*^2^ was used to measure the heterogeneity for each outcome (Higgins et al., 2003). Effect combination was performed if at least two studies reported available subgroup results. We used the random effects model to synthesize the relationship between exposure and outcomes (Lau et al., 1997). We used dichotomous variables as indicators of occupational noise exposure for meta-analysis, and used dose-response meta-analysis for residential noise exposure. Different studies categorized exposure levels differently, and the most comparable thresholds were chosen to ensure consistency of exposure levels overall. In the final included studies, different covariates were also made. Using sensitivity analysis, we further discussed the heterogeneity of the included studies. Statistical significance was determined by a *P*-value < 0.05.

## Results

### Identification of relevant studies

**Figure 1:**
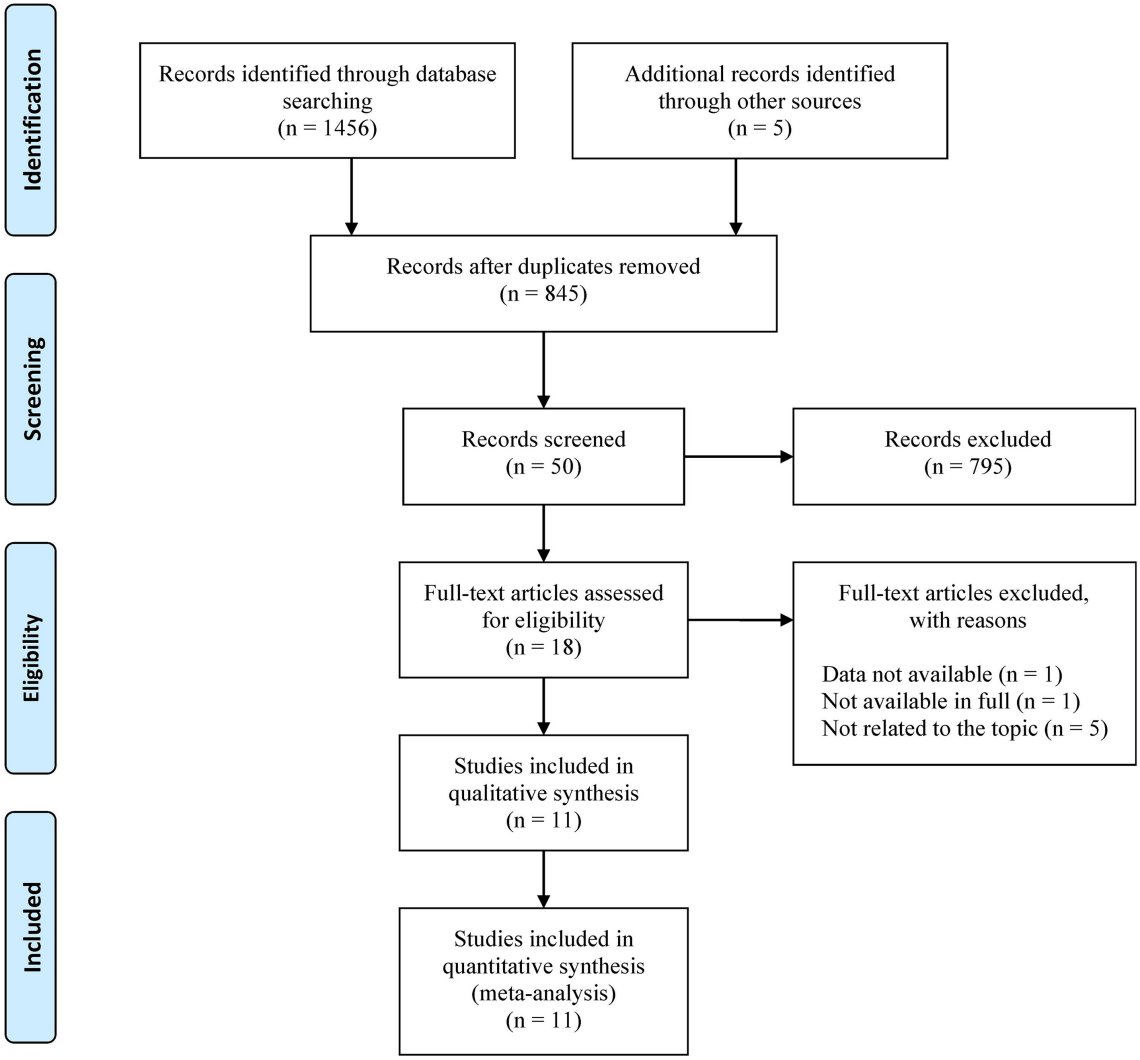
Flowchart for the systematic literature search.

A total of 1,456 results were obtained from the database search. Following the retrieval of literature on noise and pregnancy outcomes, five additional studies were added for a total of 1,461 study records. After deduplication, 616 studies were excluded, followed by 827 studies after preliminary screening. The remaining 18 studies were subjected to additional screening, including full-text readings and Supplementary materials reviews. After reading full texts, one was excluded because it was not available in full (Hartikainen-Sorri et al., 1991), one was excluded because its data was not available (Bilenko et al., 2022), and five were excluded because it was not directly related to the research topic (Bendokiene et al., 2011; van den Hooven et al., 2011; Liao et al., 2019; Costello et al., 2022; Wing et al., 2022). Supplementary Appendix B described the specifics of these excluded studies. Finally, eleven studies were included. The retrieval process was depicted in Figure 1.

### Description of studies included in the final analysis

The essential characteristics of the studies finally included were shown in Table 1. The studies included nine cohort studies and two case-control studies. The study spanned the years 1976–2017. There were six studies conducted in Europe, two in Canada, two in the United States, and one in South Korea. The majority of studies had precise case counts and sample sizes for each group, and two used continuous increments (Pedersen et al., 2017b,c). The largest study involved 1, 087, 944 deliveries (Lissåker et al., 2021), and the total cumulative sample size was 2, 092, 076. Six studies on the impact of noise exposure in residential buildings were conducted (Min and Min, 2017; Pedersen et al., 2017b,c; Auger et al., 2018; Sears et al., 2018; Thacher et al., 2021), all of which estimated noise exposure levels using databases or regression models. Among them, three studies were converted into continuous dose exposure data (Min and Min, 2017; Auger et al., 2018; Thacher et al., 2021). The other five studies focused on noise exposure in the workplace (Nurminen and Kurppa, 1989; Irwin et al., 1994; Wergeland and Strand, 1997; Haelterman et al., 2007; Lissåker et al., 2021), assessing noise exposure levels *via* field measurements or questionnaires. For both residential and occupational noise exposure, the majority of studies defined noise exposure as 50–85 dB(A), and only one study defined noise exposure as 35 dB(A) or higher (Sears et al., 2018). Five studies on occupational noise addressed HDP (Nurminen and Kurppa, 1989; Irwin et al., 1994; Wergeland and Strand, 1997; Haelterman et al., 2007; Lissåker et al., 2021), and one study addressed GDM (Lissåker et al., 2021). Four of these studies further analyzed preeclampsia as a subgroup of HDP (Irwin et al., 1994; Wergeland and Strand, 1997; Haelterman et al., 2007; Lissåker et al., 2021). There were three studies on HDP (Pedersen et al., 2017b; Auger et al., 2018; Sears et al., 2018) and GDM (Min and Min, 2017; Pedersen et al., 2017c; Thacher et al., 2021) each, and two of these articles analyzed preeclampsia separately (Pedersen et al., 2017b; Auger et al., 2018). The prevalence of HDP, preeclampsia and GDM were 2.12–8.64, 2.18–5.17, and 0.90–8.08%, respectively. Regarding adjustments for related confounders, each study differed slightly.

**Table 1:** Characteristics of studies on the relationship between noise exposure during pregnancy and pregnancy complications.

**References**	**Study period**	**Study location**	**Study design**	**Sample size**	**Exposure**		**Study outcomes**	**Covariates**
					**Level**	**Type**		
Auger et al. (2018)	2000–2013	Canada	Retrospective cohort	269,263	Per 10 dB(A)^*^	Residential	PE: 1.01 [0.96, 1.07]	Air pollution, walkability, age, parity, multiple pregnancies, comorbidity, socioeconomic deprivation, and year of pregnancy
Haelterman et al. (2007)	1997–1999	Canada	Case-control	4,582	Speak loudly^†^	Occupational	HDP: 0.96 [0.65, 1.41]	Age, parity, history of abortion, level of education, BMI, smoking, leisure-time physical activity
							PE: 0.98 [0.57, 1.68]	
Irwin et al. (1994)	1987–1989	U.S. Navy	Retrospective cohort	5,522	≥84 dB(A)	Occupational	HDP: 1.03 [0.83, 1.28]	None
							PE: 1.06 [0.76, 1.48]	
Lissåker et al. (2021)	1994–2014	Sweden	Prospective cohort	1,087,944	≥80 dB(A)	Occupational	HDP: 1.04 [1.01, 1.07]	Age, smoking, education, country of birth, particles, physical load, job strain, and exposure to low temperatures
							PE: 1.07 [1.04, 1.10]	
							GDM: 0.94 [0.88, 1.00]	
Min and Min (2017)	2002–2013	Korea	Prospective cohort	18,165	Per 10 dB(A)^*^	Residential	GDM: 1.10 [1.05, 1.16]	Age, household income relative to the median, residence area, smoking history, exercise, alcohol drinking, blood glucose levels at pre-pregnancy, and BMI
Nurminen and Kurppa (1989)	1976–1982	Finland	Retrospective cohort	1,040	≥80 dB(A)	Occupational	HDP: 1.76 [0.99, 3.12]	Maternal age, parity, the outcome of previous pregnancies, alcohol intake, and smoking
Pedersen et al. (2017b)	1996–2002	Denmark	Prospective cohort	72,745	Per 10 dB(A)	Residential	HDP: 1.08 [1.02, 1.14]	Maternal age, parity, pre-pregnancy BMI, height, disposable income, education, and season of conception
							PE: 1.10 [1.02, 1.18]	
Pedersen et al. (2017c) (2)	1996–2002	Denmark	Prospective cohort	72,745	Per 10 dB(A)	Residential	GDM: 1.15 [0.94, 1.42]	Maternal age, parity, pre-pregnancy BMI, height, disposable income, education, and season of conception
Sears et al. (2018)	2003–2006	U.S.A.	Prospective cohort	370	≥35 dB(A)	Residential	HDP: 1.32 [0.70, 2.48]	Maternal age, cotinine level, BMI, gestational age, income, education, season, race, neighborhood socioeconomic status
Thacher et al. (2021)	2004–2017	Denmark	Retrospective cohort	629,254	Per 10 dB(A)^*^	Residential	GDM: 1.01 [0.98, 1.03]	Maternal age, parity, calendar year, civil status, income, country of origin, occupational status, education level, and area-level socioeconomic variables
Wergeland and Strand (1997)	1989–1989	Norway	Case-control	3,192	≥80 dB(A)	Occupational	PE: 1.39 [1.02, 1.89]	Age, Parity, height, BMI, education, smoking in pregnancy, coffee consumption, paid work, hours of housework, father's education

* After dose effect conversion.

† To be heard by someone at 2 meters: could speak normally (control), had to speak loudly or shout (exposed).

BMI, body mass index; GDM, gestational diabetes mellitus; HDP, hypertensive disorders of pregnancy; PE, preeclampsia.

### Risk of bias

**Figure 2:**
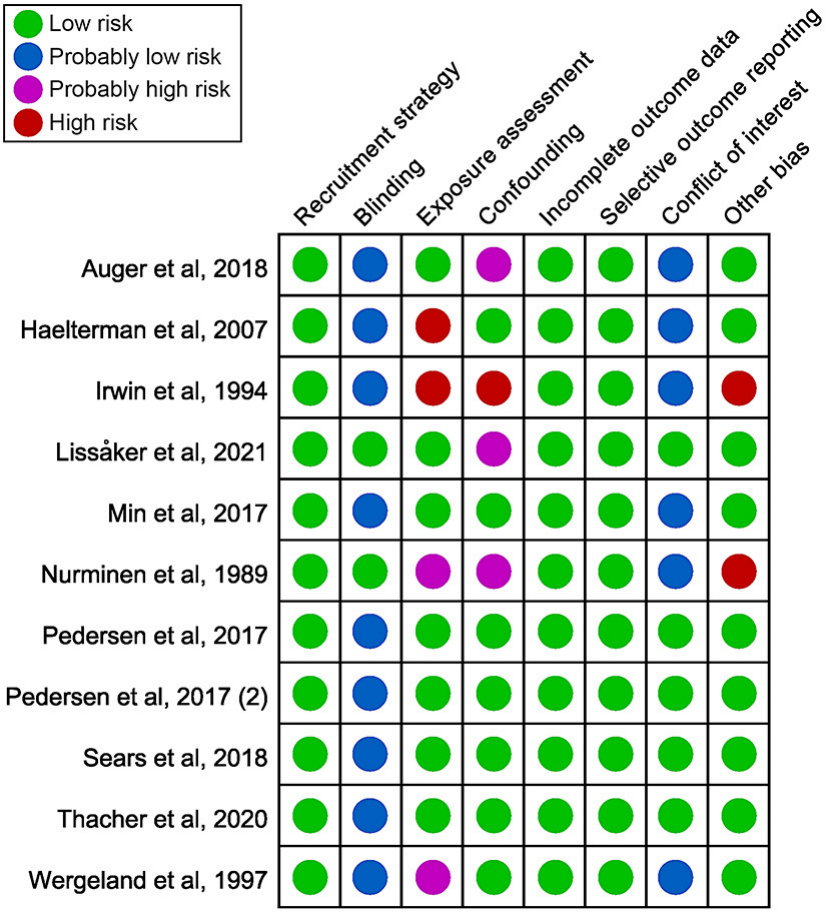
Summary of the risk of bias judgments for each included study.

Figure 2 provided a summary of the bias risk for each included study. Two studies were deemed to have a high risk of bias in exposure assessment, one because there was no quantifiable level of exposure (Haelterman et al., 2007), and the other because exposure assessment method was not presented (Irwin et al., 1994). Due to ambiguous noise measurement techniques, two studies were considered probably high risk (Nurminen and Kurppa, 1989; Wergeland and Strand, 1997). Not all adjustments were made for major confounding factors [age and body mass index (BMI)] in four studies (Nurminen and Kurppa, 1989; Irwin et al., 1994; Auger et al., 2018; Lissåker et al., 2021). Two studies were assessed as high risk in the other bias because participants were from non-general populations. They were mothers of infants with structural birth defects (Nurminen and Kurppa, 1989) and Navy enlisted personnel admitted for singleton infant delivery to military hospitals (Irwin et al., 1994). Therefore, the representativeness of the sample population could not be confirmed. Supplementary Appendix C displayed the specifics of the bias evaluation.

Some studies reported protective effects, while the majority reported harmful effects of noise exposure on pregnancy complications. The number of studies included was insufficient to evaluate publication bias. It was important to note that several studies had reported a single to multiple pregnancy complication outcomes. It was unclear whether certain complications had been evaluated but not reported, so it was possible that selective reporting occurred.

### Association of occupational noise exposure with pregnancy complications

**Figure 3:**
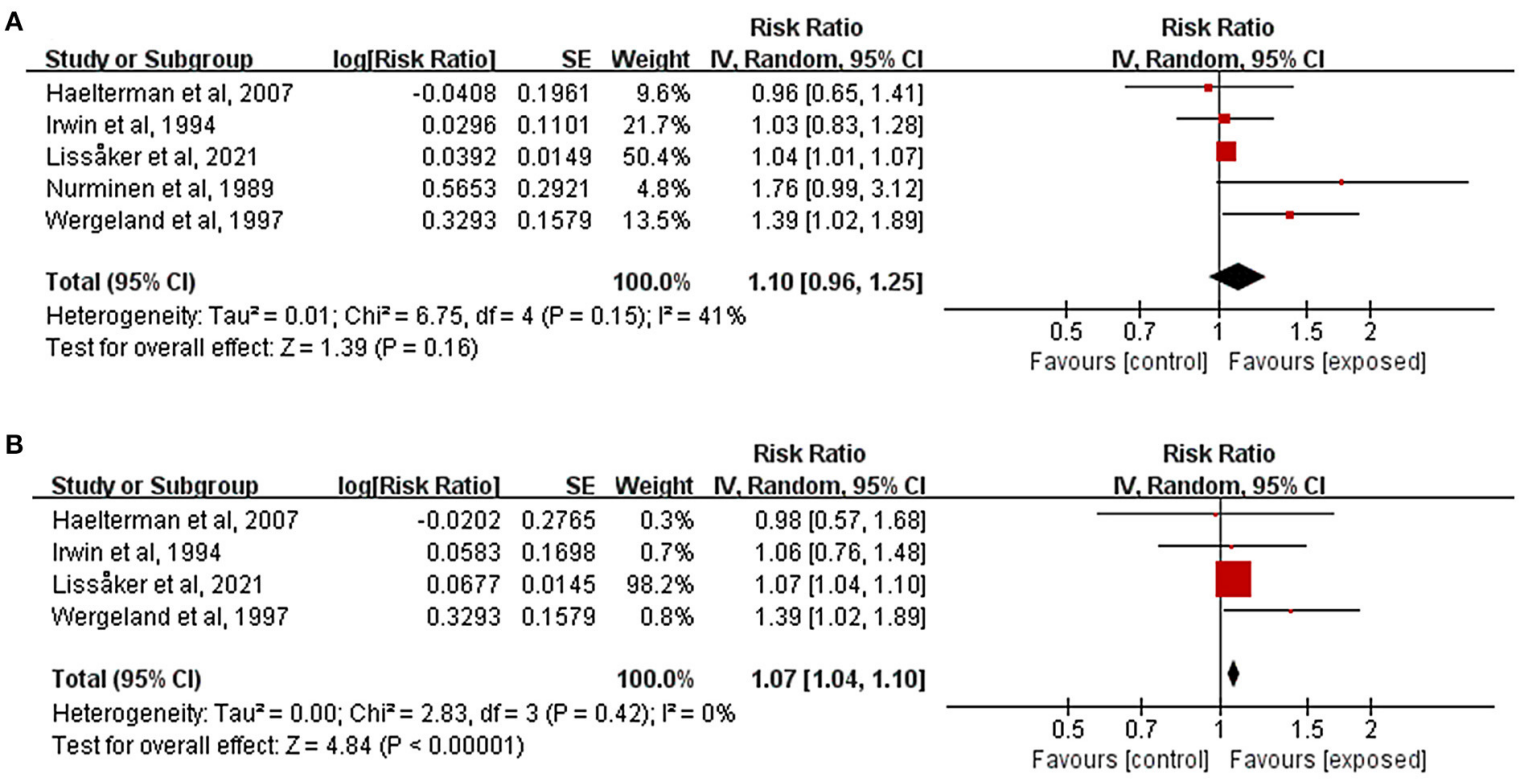
Forest plots for the relationship between occupational noise exposure and pregnancy complications. Tau^2^, Chi^2^, and df are the statistics in the heterogeneity test, and *I*^2^ is the effect variable attributable to heterogeneity. *Z*-value is the statistical variable of the overall effect, and inverse variance (IV) is the effect estimation of the study. Effect sizes are given by 95% confidence intervals (95%*CI*). The weights are determined by random effects analysis. The size of the boxes around the points reflects the weight assigned to each study. **(A)** Hypertensive disorders of pregnancy; **(B)** Preeclampsia.

Figure 3A showed estimates of the individual and combined effects of occupational noise exposure during pregnancy associated with HDP. The test for heterogeneity revealed *I*^2^ = 41% (*P* = 0.15). The results indicated that occupational noise had no significant positive effect on the incidence of HDP (*RR* = 1.10, 95%*CI*: 0.96, 1.25). Figure 3B depicted the effect sizes from four studies that evaluated the association between occupational noise exposure and the risk of preeclampsia. Exposure to occupational noise during pregnancy increased the risk of preeclampsia significantly (*RR* = 1.07, 95%*CI*: 1.04, 1.10). There was only one study that examined the association between occupational noise exposure and GDM (Lissåker et al., 2021), and the result showed that there was no significant relationship between occupational noise exposure and GDM (*RR* = 0.94, 95%*CI*: 0.88, 1.00).

### Association of residential noise exposure with pregnancy complications

**Figure 4:**
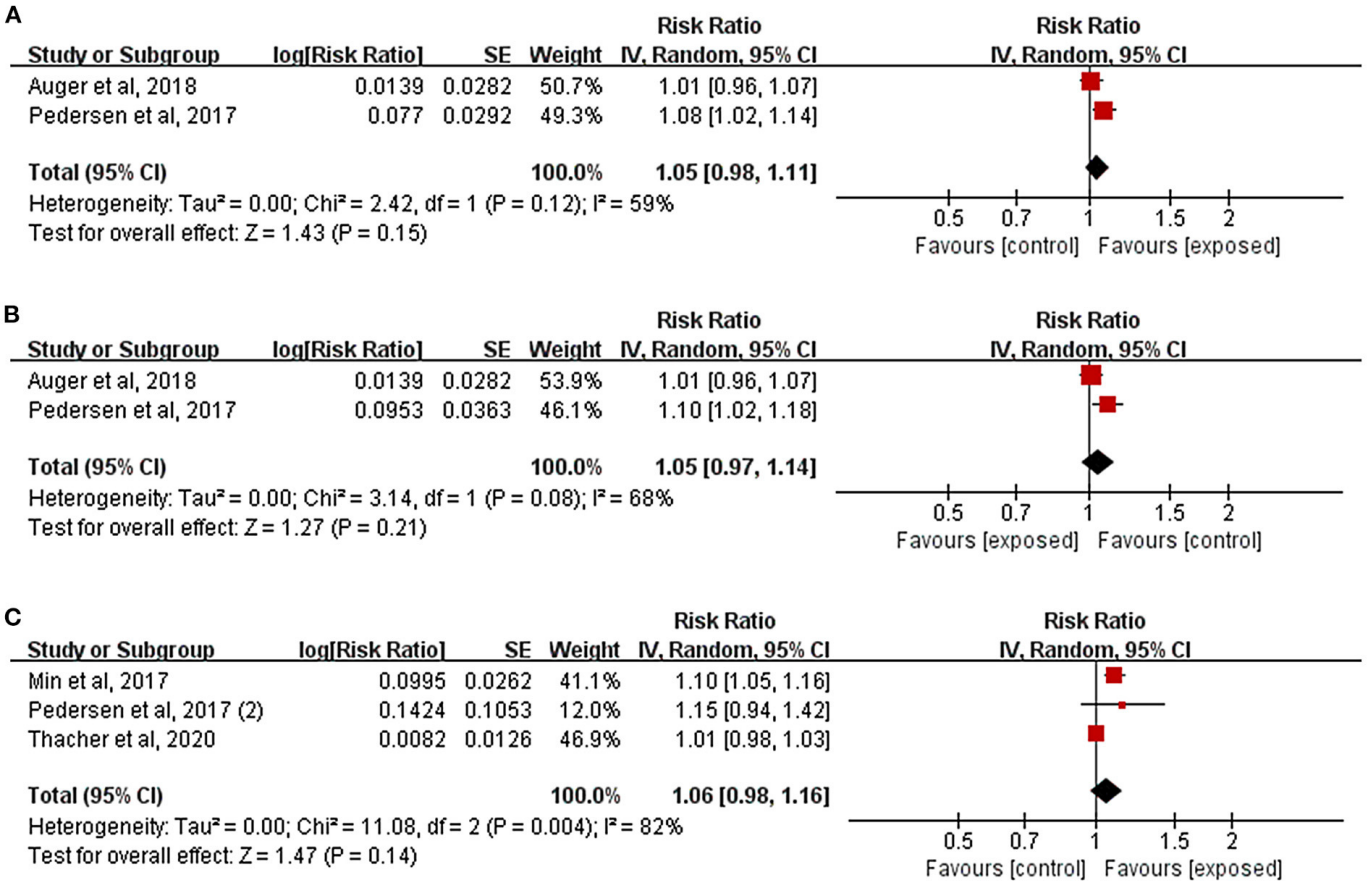
Forest plots for the relationship between residential noise exposure and pregnancy complications. Tau^2^, Chi^2^, and df are the statistics in the heterogeneity test, and *I*^2^ is the effect variable attributable to heterogeneity. *Z*-value is the statistical variable of the overall effect, and inverse variance (IV) is the effect estimation of the study. Effect sizes are given by 95% confidence intervals (95%*CI*). The weights are determined by random effects analysis. The size of the boxes around the points reflects the weight assigned to each study. **(A)** Hypertensive disorders of pregnancy; **(B)** Preeclampsia; **(C)** Gestational diabetes mellitus.

Figure 4A illustrated the effects of residential noise exposure during pregnancy on HDP risk. Because it used dichotomous variables to define noise exposure level, one study was excluded from effect combination (Sears et al., 2018). Other two studies were pooled (Pedersen et al., 2017b; Auger et al., 2018), and the results indicated that residential noise had no significant effect on the incidence of HDP (*RR* = 1.05, 95%*CI*: 0.98, 1.11). Similar findings were reported by Sears et al. in their study (*RR* = 1.32, 95%*CI*: 0.70, 2.48). Two studies further evaluated the relationship between residential noise exposure and preeclampsia; the results were similar to those presented in Figure 4B (*RR* = 1.05, 95%*CI*: 0.97, 1.14).

As depicted in Figure 4C, the results of the three studies combined showed that there was no clear relationship between residential noise and GDM (*RR* = 1.06, 95%*CI*: 0.98, 1.16), and the study by Lissåker et al. which focused on the relationship between occupational noise and GDM reported the same results (*RR* = 0.94, 95%*CI*: 0.88, 1.00).

### Quality and strength of evidence

We assessed the research evidence for every outcome using the GRADE approach. Table 2 summarized the quality and strength of the evidence, and Supplementary Appendices D, E provided details of the evaluation process. Initially, the criteria rated the quality of human observational studies as “moderate.” Among the six outcomes, the association between occupational noise exposure and GDM had only one study (Lissåker et al., 2021), so no meta-analysis or assessment of the quality of the evidence was performed. Two outcomes were downgraded for risk of bias, and four outcomes were downgraded for risk of inconsistency in the remaining five outcomes. The confidence intervals for all outcomes were narrow, no residual confounders were found that could significantly affect the effect size, and all searches were exhaustive. In conclusion, the quality of evidence was rated “low” for all five outcomes. In assessing the strength of evidence, it was determined that the strength of evidence was limited for one outcome, and inadequate for four outcomes. Only one outcome (occupational noise exposure and preeclampsia) was statistically significant.

**Table 2:** Summary of findings, quality of evidence, and strength of evidence for noise exposure during pregnancy and pregnancy complications.

**Outcomes**	**No. of participant (studies)**	**Quality of evidence rating**	**Strength of evidence rating**	**Comments**
**Effect of occupational noise exposure on pregnancy complications**				
HDP	1,102,280 (5 studies)	Low	Inadequate evidence of harmfulness	The strength of the evidence is inadequate. Chance, bias, and confounding cannot be ruled out with reasonable confidence. Because of the small number of included studies and the varying outcomes of these studies, the conclusion may be influenced by future studies.
Preeclampsia	1,101,240 (4 studies)	Low	Limited evidence of harmfulness	A positive relationship was observed between exposure and outcome, where chance, bias, and confounding cannot be ruled out with reasonable confidence. Because of the small number of included studies and the varying outcomes of these studies, we think that our conclusions may be influenced by the results of future studies. In conclusion, the strength of evidence is limited.
GDM	1,087,944 (1 study)	–	Inadequate evidence of harmfulness	There was only one study related to this outcome, so meta-analysis could not be performed.
**Effect of residential noise exposure on pregnancy complications**				
HDP	342,008 (2 studies)	Low	Inadequate evidence of harmfulness	The strength of the evidence is inadequate. Chance, bias, and confounding can be ruled out with reasonable confidence. Dose response analysis indicated that the risk of outcome did not increase with the increase of exposure levels. Because of the small number of included studies and the varying outcomes of these studies, the conclusion may be influenced by future studies.
Preeclampsia	342,008 (2 studies)	Low	Inadequate evidence of harmfulness	The strength of the evidence is inadequate. Chance, bias, and confounding can be ruled out with reasonable confidence. Dose response analysis indicated that the risk of outcome did not increase with the increase of exposure levels. Because of the small number of included studies and the varying outcomes of these studies, the conclusion may be influenced by future studies.
GDM	720,164 (3 studies)	Low	Inadequate evidence of harmfulness	The strength of the evidence is inadequate. Chance, bias, and confounding can be ruled out with reasonable confidence. Dose response analysis indicated that the risk of outcome did not increase with the increase of exposure levels. The available evidence includes the results of three high-quality studies, but because of the small number of included studies and the varying outcomes of these studies, the conclusion may be influenced by future studies.

GDM, gestational diabetes mellitus; HDP, hypertensive disorders of pregnancy.

### Sensitivity analysis

Only outcomes involving more than two studies underwent sensitivity analysis. In the results of occupational noise and HDP, the effect size changed from 1.10 (95%*CI*: 0.96, 1.25) to 1.18 (95%*CI*: 0.94, 1.48) when one study with significant effect was excluded (Lissåker et al., 2021), but the difference was not statistically significant. For occupational noise and preeclampsia, removing one study (Lissåker et al., 2021) changed risk from 1.07 (95%*CI*: 1.04, 1.10) to 1.19 (95%*CI*: 0.96, 1.46)—a substantial change, suggesting that the results were unstable. As for the relationship between residential noise and GDM, the risk rose from 1.06 (95%*CI*: 0.98, 1.16) to 1.11 (95%*CI*: 1.05, 1.16) when the study with the most weight was eliminated (Thacher et al., 2021). This was quite intriguing, and the combined findings of the two studies indicated a statistically significant negative effect. The remaining subgroups were not analyzed because there were too few studies and the sensitivity analysis was meaningless.

## Discussion

This meta-analysis analyzed eleven studies that investigated the correlation between noise exposure and pregnancy complications. These studies included participants with varying BMIs and lifestyle habits at various times, regions, and environments. Two included studies had two or more risks of bias, but the overall risk of bias was low and had no effect on the conclusion. We took different strategies to defining exposure for various types of noise. Five of the six studies on occupational noise used a specific cutoff value to distinguish the exposed group from the control group, preventing dose response conversion from being performed. The remaining study was then dichotomized, and the combined effect size was reported using dichotomy. Despite the fact that the majority of studies on the association between occupational noise exposure and pregnancy complications used 80 dB(A) as the cutoff between exposure and control, two studies used different cutoff values. One had a cutoff value of 84 dB(A) (Irwin et al., 1994) and the other used subjective criteria (speaking loudly) to determine the cutoff value (Haelterman et al., 2007). We considered 84 dB(A) to be close to 80 dB(A) and both to be within the criteria for mild noise exposure as defined by World Health Organization (Ezzatim et al., 2004). In addition, due to differences in noise measurement tools and methods, different studies would select the criteria for cutoff that were more applicable to their particular research situation; thus, this study was included in the merge. As for the study of Haelterman et al., the evaluation criteria were subjective, but the psychological impact of noise was comparable to that of other studies, so it was merged. We meticulously evaluated the subjective assessment risk in this study and determined it to be a high risk of bias in the exposure assessment. The majority of studies on residential noise reported results in the form of dose response, and after linearization, we combined the effect sizes reported by these studies. Unfortunately, only one study reported results by dichotomy (Sears et al., 2018), so it could not be included in the pooled analysis. The meta-analysis results indicated that both types of noise exposure during pregnancy were not significantly associated with HDP and GDM. Preeclampsia was analyzed separately because the subsequent incidence of cardiovascular disease was significantly higher in preeclampsia patients than in other HDP patients (Leon et al., 2019). Exposure to occupational noise was associated with an increased risk of preeclampsia, whereas the harmfulness of residential noise exposure was unclear. The results suggested that the adverse effect of noise on pregnant women might have more severe cardiovascular and endocrine-metabolic consequences.

The GRADE approach was utilized to assess the quality and strength of evidence. In all five outcomes the evidence was of low quality. The strength of evidence was limited in one outcome and inadequate in other four outcomes. Sensitivity analysis revealed that our meta-analysis combination was not entirely stable. Significant changes in effect size and heterogeneity were observed when the included study with the most significant effect was eliminated on two outcomes (occupational noise exposure and preeclampsia; residential noise exposure and GDM). In the study examining the association between occupational noise exposure and preeclampsia, Lissåker et al.'s study accounted for up to 98.2% of the total weight, and its influence on the post-combination effect was therefore substantial. Thus, after pooling, the remaining three small studies' effect sizes were underrepresented. As the study by Lissåker et al. was a national cohort study with strong evidence and narrow confidence intervals, we considered the effect size to be somewhat generalizable. After removing the Thacher et al. study from the residential noise exposure and GDM outcome, the weights of the remaining two studies in the combination were very different, that was due to the fact that there were only three studies in this outcome, and the removal of even one would have significantly altered the effect size.

Dzhambov et al. (2014) conducted a meta-analysis on the association between noise exposure and birth outcomes and fetal development in 2014. Their findings indicated that occupational noise increased the risk of HDP, confirming the findings of this study. Compared to the study by Dzhambov et al., our study comprised nine high-quality articles, seven of which were published between 2017 and 2021, three of which were large-scale national cohort studies, and we classified and further analyzed the noise in various noise type groups. Cohort studies such as those of Min et al. demonstrated that residential noise exposure increased the risk of GDM (Min and Min, 2017), which contradicted our findings. We believed this was due to the classification method of noise exposure used in the study, as Min et al. utilized a quartile classification method. In contrast, other studies classified noise according to specific decibel values (Huang et al., 2019).

Complex mechanisms underlie the association between noise exposure during pregnancy and the risk of pregnancy complications. Previous researchers have found that the risk of noise-induced cardiovascular disease is greater in women and have even suggested that increased noise sensitivity should be viewed as an independent risk factor for cardiovascular disease in women (Bluhm et al., 2007; Jarup et al., 2008; Heinonen-Guzejev et al., 2013). Due to rapid physiological changes in respiration, sympathetic nervous system activity, circulation, cardiac output, etc., during the first half of pregnancy, pregnant women are more susceptible to noise, which can result in changes to the circulatory system and endocrine dysfunction, which can then lead to pregnancy complications (Pedersen et al., 2014; Sanghavi and Rutherford, 2014). The activation of the amygdala, a portion of the cortical margin, and the hypothalamus in response to environmental noise leads to the stimulation of the vagal-adrenal axis and the release of catecholamines (de Weerth and Buitelaar, 2005; Recio et al., 2016; Nieuwenhuijsen et al., 2017; Munzel et al., 2018). Additionally, noise stress directly increases cytokine levels, causing endothelium damage (Pedersen et al., 2014; Halim and Halim, 2019; Herzog et al., 2019). Moreover, noise-induced sleep disorders can disrupt the endocrine and metabolic systems, leading to abnormal glucose metabolism (Ising and Kruppa, 2004; Twedt et al., 2015; McHill and Wright, 2017). These mechanisms may interact to cause complications during pregnancy.

Our research had a few limitations. First, because there was so little research on this topic, our analysis only included eleven study records. In the studies on occupational noise exposure included in this analysis, the noise evaluation criteria were primarily judged based on the sound intensity over a brief period, with the persistence of noise exposure and the cumulative effect of dose over time being ignored. Our study analyzed occupational noise exposure using dichotomous variables as opposed to continuous variables, so there was no dose effect. In addition, the majority of included studies did not consider modifying noise protection measures, such as the use of earmuffs to prevent occupational noise or acoustic glass to reduce residential noise. Moreover, when assessing noise exposure in residential areas, only road traffic noise was considered, while noise from community environments and commercial establishments was largely ignored.

There were also three discrepancies between the actual review and protocol. In the protocol, we stated that the research deadline was 2021.5.31, but because the research period was extended, we used 2022.9.30 as the search deadline. In addition, the CNKI and WANFANG databases that were originally intended to be included were omitted in favor of Ovid and Cochrane Library. Protocol was followed for the retrieval of PubMed, Web Of Science, Embase, and Scopus. Importantly, we focused on the association between noise and pregnancy complications without classifying noise in the protocol. During the review process, we shifted the focus to the association between different types of noise and pregnancy complications.

To the best of our knowledge, no previous meta-analysis had examined the association between different types of noise exposure and pregnancy complications; therefore, our study filled this void. Overall, the evidence suggested that occupational noise exposure during pregnancy was associated with an increased risk of preeclampsia. Our research confirmed that the harmful effects of noise exposure were related to both the mode and level of exposure.

In this field, extensive cohort studies are still rare. Numerous studies have speculated about the mechanisms by which noise affects pregnant women and fetuses, but no mechanism has been conclusively demonstrated. Vibration also obscures the effect of noise as a mechanical wave on the maternal circulatory and endocrine systems. Therefore, additional multi-center, high-quality prospective cohort studies, experimental studies employing protective measures for intervention, and basic medical research (such as additional biomarker analysis of inflammatory factors, neuroendocrine stress levels, etc.) are required to enhance our understanding of the association between noise exposure and pregnancy complications. Only one of the eleven studies was conducted in Asia (Min and Min, 2017), while ten were conducted in Europe and North America. There is no research on this subject in developing nations. Developing nations have more labor-intensive industries than developed nations, and their citizens are more susceptible to occupational noise and traffic noise (Shang et al., 2020; Xie et al., 2021). Therefore, to generalize the findings, research should be expanded to developing nations with high noise pollution levels. It is also beneficial to comprehend the effects of noise in various locations and social settings. In addition, the macro social and economic situation, working environments, jobs, and other factors should be modified, as they may contribute to a variety of mental stress issues and influence the outcome (Wang et al., 2020). Furthermore, more researches are required to combine subjective evaluation and objective measurement in order to assess the impact of noise on everyone more accurately.

## Conclusions

Occupational noise exposure was related with preeclampsia, but there was no indication of a detrimental effect of noise exposure during pregnancy on HDP or GDM. This study provides evidence for further exploration of the association between exposure to environmental noise and pregnancy complications.

## Data availability statement

The original contributions presented in the study are included in the article/Supplementary material, further inquiries can be directed to the corresponding author.

## Author contributions

ZW and RQ: formal analysis, methodology, writing—original draft, and visualization. WX: formal analysis and methodology. LS: methodology, writing—review and editing, and funding acquisition. MX, BZ, and LY: investigation and writing—review and editing. SZ and LZ: investigation. WY: conceptualization, writing—review and editing, supervision, and funding acquisition. All authors contributed to the article and approved the submitted version.

## Funding

The present study was supported by National Social Science Foundation of China (Grant numbers 20BRK037).

## Conflict of interest

The authors declare that the research was conducted in the absence of any commercial or financial relationships that could be construed as a potential conflict of interest.

## Publisher's note

All claims expressed in this article are solely those of the authors and do not necessarily represent those of their affiliated organizations, or those of the publisher, the editors and the reviewers. Any product that may be evaluated in this article, or claim that may be made by its manufacturer, is not guaranteed or endorsed by the publisher.

## Abbreviations

BMI: body mass index
GDM: gestational diabetes mellitus
GRADE: grading of recommendations assessment development and evaluation
HDP: hypertensive disorders of pregnancy
HELLP: hemolysis, elevated liver enzymes and low platelets
ICD: international classification of diseases.
