# Supplementary material for: Association between noise exposure during pregnancy and pregnancy complications: A meta-analysis
Source: Front Psychol. 2022 Nov 21;13:1026996. doi: 10.3389/fpsyg.2022.1026996 (PMC9721198; doi:10.3389/fpsyg.2022.1026996)
Supplement: Supplementary file 1 [file Data_Sheet_1.docx]

**Appendix A: Search strategies**

| **#** | **Query** |
| --- | --- |
| #1 | Pregnan* OR Gestat* OR Impregnat* OR Cyes* |
| #2 | Hypertensi* OR ("High blood pressure") OR HBP |
| #3 | Diabet* OR Mellitus* OR Hyperglyc* |
| #4 | Eclampsia* OR Preeclampsia* OR HDP OR HELLP OR GDM |
| #5 | Nois* OR Decibel* OR Voic* OR Sound* OR Loud* |
| #6 | Epidemi* OR Observ* or Cohort* OR "Follow-up" or Panel* or "Cross-sectional" or Longitud* or Retrospect* or ("Case control") or Prevalen* |
| #7 | Stud* or Analys* |
| #8 | #2 OR #3 |
| #9 | #1 AND #8 |
| #10 | #4 OR #9 |
| #11 | #5 AND #10 |
| #12 | #6 AND #7 |
| #13 | #11 AND #12 |
| Filters | Full text, Humans, English, Original and review article. |

**Pubmed:** 129 results.

(((((Pregnan*[All fields] OR Gestat*[All fields] OR Impregnat*[All fields] OR Cyes*[All fields]) AND ((Hypertensi*[All fields] OR ("High blood pressure") OR HBP) OR (Diabet*[All fields] OR Mellitus*[All fields] OR Hyperglyc*[All fields]))) OR Eclampsia* [All fields] OR Preeclampsia* [All fields] OR HDP OR HELLP OR GDM) AND (Nois*[All fields] OR Decibel*[All fields] OR Voic*[All fields] OR Sound*[All fields] OR Loud*[All fields])) AND ((Epidemi*[All fields] or Observ*[All fields] or Cohort*[All fields] or ("Follow-up") or Panel*[All fields] or ("Cross-sectional") or Longitud*[All fields] or Retrospect*[All fields] or ("Case control") or Prevalen*[All fields]) and (Stud*[All fields] or Analys*[All fields]))) Filters: Full text, Humans, English

**Web Of Science:** 159 results.

TS=(((((Pregnan* OR Gestat* OR Impregnat* OR Cyes*) AND ((Hypertensi* OR ("High blood pressure") OR HBP) OR (Diabet* OR Mellitus* OR Hyperglyc*))) OR Eclampsia* OR Preeclampsia* OR HDP OR HELLP OR GDM) AND (Nois* OR Decibel* OR Voic* OR Sound* OR Loud*)) AND ((Epidemi* or Observ* or Cohort* or (“Follow-up”) or Panel* or (“Cross-sectional”) or Longitud* or Retrospect* or (“Case control”) or Prevalen*) and (Stud* or Analys*))) and Humans (MeSH) and English (Language) and (Article or Other or Review article or Unspecified (Document types))

**Scopus:** 183 results.

TITLE-ABS-KEY ((((((pregnan* OR gestat* OR impregnat* OR cyes*) AND ((hypertensi* OR ("High blood pressure") OR hbp) OR (diabet* OR mellitus* OR hyperglyc*))) OR eclampsia* OR preeclampsia* OR hdp OR hellp OR gdm) AND (nois* OR decibel* OR voic* OR sound* OR loud*)) AND ((epidemi* OR observ* OR cohort* OR ("Follow-up") OR panel* OR ("Cross-sectional") OR longitud* OR retrospect* OR ("Case control") OR prevalen*) AND (stud* OR analys*)))) AND (LIMIT-TO (DOCTYPE, "ar") OR LIMIT-TO (DOCTYPE, "re")) AND (LIMIT-TO (EXACTKEYWORD, "Human")) AND (LIMIT-TO (LANGUAGE, "English"))

**Embase:** 247 results.

((pregnan* OR gestat* OR impregnat* OR cyes*) AND (hypertensi* OR 'high blood pressure' OR hbp OR diabet* OR mellitus* OR hyperglyc*) OR eclampsia* OR preeclampsia* OR hdp OR hellp OR gdm) AND (nois* OR decibel* OR voic* OR sound* OR loud*) AND (epidemi* OR observ* OR cohort* OR 'follow-up' OR panel* OR 'cross-sectional' OR longitud* OR retrospect* OR 'case control' OR prevalen*) AND (stud* OR analys*) AND ([article]/lim OR [article in press]/lim OR [review]/lim) AND [english]/lim AND [humans]/lim

**Ovid:** 486 results.

((((((Pregnan* or Gestat* or Impregnat* or Cyes*) and (Hypertensi* or "High blood pressure" or HBP or Diabet* or Mellitus* or Hyperglyc*)) or Eclampsia* or Preeclampsia* or HDP or HELLP or GDM) and (Nois* or Decibel* or Voic* or Sound* or Loud*) and ((Epidemi* or Observ* or Cohort* or Follow-up or Panel* or Cross-sectional or Longitud* or Retrospect* or "Case control" or Prevalen*) and (Stud* or Analys*))).tx.) and (("Original article" or "Review article").pt,mt.) and (english.lg.))

Limit by: Full texts, Humans

**Cochrane Library:** 246 results.

(((((Pregnan* OR Gestat* OR Impregnat* OR Cyes*) AND ((Hypertensi* OR ("High blood pressure") OR HBP) OR (Diabet* OR Mellitus* OR Hyperglyc*))) OR Eclampsia* OR Preeclampsia* OR HDP OR HELLP OR GDM) AND (Nois* OR Decibel* OR Voic* OR Sound* OR Loud*)) AND ((Epidemi* or Observ* or Cohort* or (“Follow-up”) or Panel* or (“Cross-sectional”) or Longitud* or Retrospect* or (“Case control”) or Prevalen*) and (Stud* or Analys*))) in All Text - in Cochrane Reviews, Trials

**Appendix B: Excluded full texts**

We excluded 7 articles after full text screening.

1 article was not available in full.

A. L. Hartikainen-Sorri, P. Kirkinen, M. Sorri, H. Anttonen, R. Tuimala, No effect of experimental noise exposure on human pregnancy. Obstet. Gynecol. 77, 611-615 (1991).

1 article’s data was not available.

Bilenko, N., Ashin, M., Friger, M., Fischer, L., Sergienko, R., and Sheiner, E.. Traffic Noise and Ambient Air Pollution Are Risk Factorsfor Preeclampsia. Journal of Clinical Medicine 11(15) (2022).

5 articles were not related to the topic:

I. Bendokiene, R. Grazuleviciene, A. Dedele, Risk of hypertension related to road traffic noise among reproductive-age women. Noise Health 13, 371-377 (2011).

E. H. van den Hooven et al., Air Pollution, Blood Pressure, and the Risk of Hypertensive Complications During Pregnancy The Generation R Study. Hypertension 57, 406-U138 (2011).

J. Liao et al., Effect of residential exposure to green space on maternal blood glucose levels, impaired glucose tolerance, and gestational diabetes mellitus. Environ. Res. 176, (2019).

J. M. Costello, M. A. Steurer, R. J. Baer, J. S. Witte, L. L. Jelliffe-Pawlowski, Residential particulate matter, proximity to major roads, traffic density and traffic volume as risk factors for preterm birth in California. Paediatric and Perinatal Epidemiology 36, 70-79 (2022).

S. E. Wing et al., Aircraft noise and vehicle traffic-related air pollution interact to affect preterm birth risk in Los Angeles, California. Sci. Total Environ. 829, (2022).

**Appendix C: Characteristics of risk of bias in each study**

| **Bias** | **Rating** | **Support for judgment** |
| --- | --- | --- |
| **Auger et al, 2018** |  |  |
| Recruitment strategy | Low risk | The strategy for recruiting participants was consistent across study groups. |
| Blinding | Probably low risk | The authors did not discuss blinding but the study design prevents knowledge of exposure groups. |
| Exposure assessment | Low risk | The exposure assessment methods were robust and included a detailed description of QA/QC. |
| Confounding | Probably high risk | The study accounted for some but not all of the important potential confounders. |
| Incomplete outcome data | Low risk | The study did not have incomplete outcome data. |
| Selective outcome reporting | Low risk | The study is free of suggestion of selective outcome reporting. All of the study’s specified outcomes were adequately reported. |
| Conflict of interest | Probably low risk | Associated funds and persons appear to be from government and/or academia only and free of financial interests in study results. However, no claim denying conflicts of interest was made. |
| Other bias | Low risk | No other potential biases are suspected. |
| **Haelterman et al, 2007** |  |  |
| Recruitment strategy | Low risk | The strategy for recruiting participants was consistent across study groups. |
| Blinding | Probably low risk | The authors did not discuss blinding but the study design prevents knowledge of exposure groups. |
| Exposure assessment | High risk | The direct exposure measurement was not used, and had no quantitive level of exposure. |
| Confounding | Low risk | The study accounted for all important potential confounders. |
| Incomplete outcome data | Low risk | The study did not have incomplete outcome data. |
| Selective outcome reporting | Low risk | The study is free of suggestion of selective outcome reporting. All of the study’s specified outcomes were adequately reported. |
| Conflict of interest | Probably low risk | Associated funds and persons appear to be from government and/or academia only and free of financial interests in study results. However, no claim denying conflicts of interest was made. |
| Other bias | Low risk | No other potential biases are suspected. |
| **Irwin et al, 1994** |  |  |
| Recruitment strategy | Low risk | The strategy for recruiting participants was consistent across study groups. |
| Blinding | Probably low risk | The authors did not discuss blinding but the study design prevents knowledge of exposure groups. |
| Exposure assessment | High risk | The study did not show exposure assessment methods. |
| Confounding | High risk | The study did not account for or evaluate important potential confounders. |
| Incomplete outcome data | Low risk | The proportion of missing outcomes compared with observed event risk not enough to have a biologically relevant impact on the intervention effect estimate. |
| Selective outcome reporting | Low risk | The study is free of suggestion of selective outcome reporting. All of the study’s specified outcomes were adequately reported. |
| Conflict of interest | Probably low risk | No claim denying conflicts of interest and associated funds and persons were made, but otherwise no reason to suspect there was conflict of interest. |
| Other bias | High risk | Because the study population was selected from navy women, the study could be biased towards inclusion of high-risk pregnancies. |
| **Lissåker et al, 2021** |  |  |
| Recruitment strategy | Low risk | The strategy for recruiting participants was consistent across study groups. |
| Blinding | Low risk | Knowledge of the exposure groups were adequately prevented during the study. |
| Exposure assessment | Low risk | The exposure assessment methods were robust and included a detailed description of QA/QC. |
| Confounding | Probably high risk | The study accounted for some but not all of the important potential confounders. |
| Incomplete outcome data | Low risk | The study did not have incomplete outcome data. |
| Selective outcome reporting | Low risk | The study is free of suggestion of selective outcome reporting. All of the study’s specified outcomes were adequately reported. |
| Conflict of interest | Low risk | The authors report no conflict of interest, and associated funds and persons appear to be from government and/or academia only. |
| Other bias | Low risk | No other potential biases are suspected. |
| **Min et al, 2017** |  |  |
| Recruitment strategy | Low risk | The strategy for recruiting participants was consistent across study groups. |
| Blinding | Probably low risk | The authors did not discuss blinding but the study design prevents knowledge of exposure groups. |
| Exposure assessment | Low risk | The exposure assessment methods were robust and included a detailed description of QA/QC. |
| Confounding | Low risk | The study accounted for all important potential confounders. |
| Incomplete outcome data | Low risk | The study did not have incomplete outcome data. |
| Selective outcome reporting | Low risk | The study is free of suggestion of selective outcome reporting. All of the study’s specified outcomes were adequately reported. |
| Conflict of interest | Probably low risk | Associated funds and persons appear to be from government and/or academia only and free of financial interests in study results. However, no claim denying conflicts of interest was made. |
| Other bias | Low risk | No other potential biases are suspected. |
| **Nurminen et al, 1989** |  |  |
| Recruitment strategy | Low risk | The strategy for recruiting participants was consistent across study groups. |
| Blinding | Low risk | Knowledge of the exposure groups were adequately prevented during the study. |
| Exposure assessment | Probably high risk | The study reported specific exposure measures in citations, but citations could not be retrieved. |
| Confounding | Probably high risk | The study accounted for some but not all of the important potential confounders. |
| Incomplete outcome data | Low risk | The proportion of missing outcomes compared with observed event risk not enough to have a biologically relevant impact on the intervention effect estimate. |
| Selective outcome reporting | Low risk | The study is free of suggestion of selective outcome reporting. All of the study’s specified outcomes were adequately reported. |
| Conflict of interest | Probably low risk | Associated funds and persons appear to be from government and/or academia only and free of financial interests in study results. However, no claim denying conflicts of interest was made. |
| Other bias | High risk | Because the study population was selected from mothers of infants with structural birth defects, the study could be biased towards inclusion of high-risk pregnancies. |
| **Pedersen et al, 2017** |  |  |
| Recruitment strategy | Low risk | The strategy for recruiting participants was consistent across study groups. |
| Blinding | Probably low risk | The authors did not discuss blinding but the study design prevents knowledge of exposure groups. |
| Exposure assessment | Low risk | The exposure assessment methods were robust and included a detailed description of QA/QC. |
| Confounding | Low risk | The study accounted for all important potential confounders. |
| Incomplete outcome data | Low risk | The study did not have incomplete outcome data. |
| Selective outcome reporting | Low risk | The study is free of suggestion of selective outcome reporting. All of the study’s specified outcomes were adequately reported. |
| Conflict of interest | Low risk | The authors report no conflict of interest, and associated funds and persons appear to be from government and/or academia only. |
| Other bias | Low risk | No other potential biases are suspected. |
| **Pedersen et al, 2017 (2)** |  |  |
| Recruitment strategy | Low risk | The strategy for recruiting participants was consistent across study groups. |
| Blinding | Probably low risk | The authors did not discuss blinding but the study design prevents knowledge of exposure groups. |
| Exposure assessment | Low risk | The exposure assessment methods were robust and included a detailed description of QA/QC. |
| Confounding | Low risk | The study accounted for all important potential confounders. |
| Incomplete outcome data | Low risk | The study did not have incomplete outcome data. |
| Selective outcome reporting | Low risk | The study is free of suggestion of selective outcome reporting. All of the study’s specified outcomes were adequately reported. |
| Conflict of interest | Low risk | The authors report no conflict of interest, and associated funds and persons appear to be from government and/or academia only. |
| Other bias | Low risk | No other potential biases are suspected. |
| **Sears et al, 2018** |  |  |
| Recruitment strategy | Low risk | The strategy for recruiting participants was consistent across study groups. |
| Blinding | Probably low risk | The authors did not discuss blinding but the study design prevents knowledge of exposure groups. |
| Exposure assessment | Low risk | The exposure assessment methods were robust and included a detailed description of QA/QC. |
| Confounding | Low risk | The study accounted for all important potential confounders. |
| Incomplete outcome data | Low risk | The study did not have incomplete outcome data. |
| Selective outcome reporting | Low risk | The study is free of suggestion of selective outcome reporting. All of the study’s specified outcomes were adequately reported. |
| Conflict of interest | Low risk | The authors report no conflict of interest, and associated funds and persons appear to be from government and/or academia only. |
| Other bias | Low risk | No other potential biases are suspected. |
| **Thacher et al, 2020** |  |  |
| Recruitment strategy | Low risk | The strategy for recruiting participants was consistent across study groups. |
| Blinding | Probably low risk | The authors did not discuss blinding but the study design prevents knowledge of exposure groups. |
| Exposure assessment | Low risk | The exposure assessment methods were robust and included a detailed description of QA/QC. |
| Confounding | Low risk | The study accounted for some important potential confounders, and reported some other potential confounders were evaluated and omitted because inclusion did not substantially affect the results. |
| Incomplete outcome data | Low risk | The study did not have incomplete outcome data. |
| Selective outcome reporting | Low risk | The study is free of suggestion of selective outcome reporting. All of the study’s specified outcomes were adequately reported. |
| Conflict of interest | Low risk | The authors report no conflict of interest, and associated funds and persons appear to be from government and/or academia only. |
| Other bias | Low risk | No other potential biases are suspected. |
| **Wergeland, 1997** |  |  |
| Recruitment strategy | Low risk | The strategy for recruiting participants was consistent across study groups. |
| Blinding | Probably low risk | The authors did not discuss blinding but the study design prevents knowledge of exposure groups. |
| Exposure assessment | Probably high risk | The study reported specific exposure measures in citations, but citations could not be retrieved. |
| Confounding | Low risk | The study accounted for all important potential confounders. |
| Incomplete outcome data | Low risk | The proportion of missing outcomes compared with observed event risk not enough to have a biologically relevant impact on the intervention effect estimate. |
| Selective outcome reporting | Low risk | The study is free of suggestion of selective outcome reporting. All of the study’s specified outcomes were adequately reported. |
| Conflict of interest | Probably low risk | Associated funds and persons appear to be from government and/or academia only and free of financial interests in study results. However, no claim denying conflicts of interest was made. |
| Other bias | Low risk | No other potential biases are suspected. |

**Appendix D: Evidence quality of each outcome**

| **Factor** | **Rating** | **Basis** |
| --- | --- | --- |
| **Occupational noise to HDP** |  |  |
| **Initial rating** | Moderate |  |
| **Downgrade factors** |  |  |
| Risk of bias across studies | -1 | There were some substantial risks of bias across the body of available evidence. Two studies both have high / probably high risks on exposure assessment, confounding, and other bias (the study population is not representative). Additionally, the other three studies also have probably high risks on exposure assessment or confounding. |
| Indirectness | 0 | The studies assessed population, exposure, comparators, and outcome of interest. |
| Inconsistency | 0 | All studies used dichotomies to define noise exposure levels. The result of the meta-analysis for the relationship between occupational noise and HDP did not appear to be strongly influenced by an individual study. |
| Imprecision | 0 | The confidence interval of the meta-analysis for the risk of HDP is sufficiently narrow. |
| Publication bias | 0 | We found no reason to suspect publication bias. The search was comprehensive. |
| **Upgrade factors** |  |  |
| Large magnitude of effect | 0 | We did not consider the estimated effects large. |
| Dose response | 0 | Only one study was modeled by categorized incremental exposure and showed evidence of a dose-response relationship, it was obvious that the evidence was not compelling enough for an upgrade. |
| Confounding minimizes effect | 0 | We did not find evidence to suggest that possible residual confounders or biases would reduce the effect estimate. |
| **Overall grade** | 0 |  |
| **Resulting rating** | Low | Moderate + (-1) = Low. |
| **Occupational noise to preeclampsia** |  |  |
| **Initial rating** | Moderate |  |
| **Downgrade factors** |  |  |
| Risk of bias across studies | -1 | There were some substantial risks of bias across the body of available evidence. One study has high / probably high risks on exposure assessment, confounding, and other bias (the study population is not representative). The other three studies also have probably high risks on exposure assessment or confounding. |
| Indirectness | 0 | The studies assessed population, exposure, comparators, and outcome of interest. |
| Inconsistency | -1 | All studies used dichotomies to define noise exposure levels. The result of the meta-analysis for the relationship between occupational noise and preeclampsia was strongly influenced by an individual study. (Lissåker et al. 2021). |
| Imprecision | 0 | The confidence interval of the meta-analysis for the risk of preeclampsia is sufficiently narrow. |
| Publication bias | 0 | We found no reason to suspect publication bias. The search was comprehensive. |
| **Upgrade factors** |  |  |
| Large magnitude of effect | 0 | We did not consider the estimated effects large. |
| Dose response | 0 | Only one study was modeled by categorized incremental exposure and showed evidence of a dose-response relationship, it was obvious that the evidence was not compelling enough for an upgrade. |
| Confounding minimizes effect | 0 | We did not find evidence to suggest that possible residual confounders or biases would reduce the effect estimate. |
| **Overall grade** | 0 |  |
| **Resulting rating** | Low | Moderate + (-2) = Low. |
| **Residential noise to HDP** |  |  |
| **Initial rating** | Moderate |  |
| **Downgrade factors** |  |  |
| Risk of bias across studies | 0 | There is no indication that there is a substantial risk of bias across the body of available evidence, particularly for the studies included in the meta-analysis. |
| Indirectness | 0 | The studies assessed population, exposure, comparators, and outcome of interest. |
| Inconsistency | -2 | All studies used continuous variables to define noise exposure. The result of the meta-analysis for the relationship between residential noise and HDP was strongly influenced by each study, and a new study might influence the result. |
| Imprecision | 0 | The confidence intervals of the meta-analysis for the risk of HDP is sufficiently narrow. |
| Publication bias | 0 | We found no reason to suspect publication bias. The search was comprehensive. |
| **Upgrade factors** |  |  |
| Large magnitude of effect | 0 | We did not consider the estimated effects large. |
| Dose response | 0 | All studies were modeled by categorized incremental exposure, but the risk of outcome did not increase with the increase of exposure levels. The evidence was not enough for an upgrade. |
| Confounding minimizes effect | 0 | We did not find evidence to suggest that possible residual confounders or biases would reduce the effect estimate. |
| **Overall grade** | 0 |  |
| **Resulting rating** | Low | Moderate + (-2) = Low. |
| **Residential noise to preeclampsia** |  |  |
| **Initial rating** | Moderate |  |
| **Downgrade factors** |  |  |
| Risk of bias across studies | 0 | There is no indication that there is a substantial risk of bias across the body of available evidence, particularly for the studies included in the meta-analysis. |
| Indirectness | 0 | The studies assessed population, exposure, comparators, and outcome of interest. |
| Inconsistency | -2 | All studies used continuous variables to define noise exposure. The result of the meta-analysis for the relationship between residential noise and preeclampsia was strongly influenced by each study, and a new study might influence the result. |
| Imprecision | 0 | The confidence intervals of the meta-analysis for the risk of preeclampsia is sufficiently narrow. |
| Publication bias | 0 | We found no reason to suspect publication bias. The search was comprehensive. |
| **Upgrade factors** |  |  |
| Large magnitude of effect | 0 | We did not consider the estimated effects large. |
| Dose response | 0 | All studies were modeled by categorized incremental exposure, but the risk of outcome did not increase with the increase of exposure levels. The evidence was not enough for an upgrade. |
| Confounding minimizes effect | 0 | We did not find evidence to suggest that possible residual confounders or biases would reduce the effect estimate. |
| **Overall grade** | 0 |  |
| **Resulting rating** | Low | Moderate + (-2) = Low. |
| **Residential noise to GDM** |  |  |
| **Initial rating** | Moderate |  |
| **Downgrade factors** |  |  |
| Risk of bias across studies | 0 | There is no indication that there is a substantial risk of bias across the body of available evidence, particularly for the studies included in the meta-analysis. |
| Indirectness | 0 | The studies assessed population, exposure, comparators, and outcome of interest. |
| Inconsistency | -1 | All studies used continuous variables to define noise exposure. The result of the meta-analysis for the relationship between residential noise and preeclampsia was strongly influenced by an individual study. |
| Imprecision | 0 | The confidence intervals of the meta-analysis for the risk of GDM is sufficiently narrow. |
| Publication bias | 0 | We found no reason to suspect publication bias. The search was comprehensive. |
| **Upgrade factors** |  |  |
| Large magnitude of effect | 0 | We did not consider the estimated effects large. |
| Dose response | 0 | All studies were modeled by categorized incremental exposure, but the risk of outcome did not increase with the increase of exposure levels. The evidence was not enough for an upgrade. |
| Confounding minimizes effect | 0 | We did not find evidence to suggest that possible residual confounders or biases would reduce the effect estimate. |
| **Overall grade** | 0 |  |
| **Resulting rating** | Low | Moderate + (-1) = Low. |

Abbreviations: GDM, gestational diabetes mellitus; HDP, hypertensive disorders of pregnancy.

**Appendix E: Evidence strength of each outcome**

| **Factor** | **Basis** |
| --- | --- |
| **Occupational noise to HDP** |  |
| Quality of body of evidence | Low |
| Direction of effect estimate | No significant relationship was observed between exposure to occupational noise and HDP. |
| Confidence in effect estimate | A new study might have an effect estimate that would make the results of the meta-analysis insignificant, because the studies we included were limited and these studies reported different results. |
| Other compelling attributes of the data that may influence certainty | None |
| Overall strength of evidence | The strength of the evidence is inadequate, that is, occupational noise exposure might influent the risk of HDP. Chance, bias, and confounding cannot be ruled out with reasonable confidence. Because of the small number of included studies and the varying outcomes of these studies, we think that our conclusions may be influenced by the results of future studies. |
| **Occupational noise to preeclampsia** |  |
| Quality of body of evidence | Low |
| Direction of effect estimate | The risk of preeclampsia increased with increasing exposure to occupational noise exposure. |
| Confidence in effect estimate | A new study might have an effect estimate that would make the results of the meta-analysis insignificant, because the studies we included were limited and these studies reported different results. |
| Other compelling attributes of the data that may influence certainty | None |
| Overall strength of evidence | A positive relationship was observed between exposure and outcome, where chance, bias, and confounding cannot be ruled out with reasonable confidence. Because of the small number of included studies and the varying outcomes of these studies, we think that our conclusions may be influenced by the results of future studies. In conclusion, the strength of evidence is limited. |
| **Residential noise to HDP** |  |
| Quality of body of evidence | Low |
| Direction of effect estimate | No significant relationship was observed between exposure to residential noise and HDP. |
| Confidence in effect estimate | A new study would likely have an effect estimate that would make the results of the meta-analysis insignificant, because the studies we included were quite limited and these studies reported quite different results. |
| Other compelling attributes of the data that may influence certainty | None |
| Overall strength of evidence | The strength of the evidence is inadequate, that is, residential noise exposure might influent the risk of HDP. Chance, bias, and confounding can be ruled out with reasonable confidence. Dose response analysis indicated that the risk of outcome did not increase with the increase of exposure levels. Moreover, because of the small number of included studies and the varying outcomes of these studies, we think that our conclusions may be influenced by the results of future studies. |
| **Residential noise to preeclampsia** |  |
| Quality of body of evidence | Low |
| Direction of effect estimate | No significant relationship was observed between exposure to residential noise and preeclampsia. |
| Confidence in effect estimate | A new study would likely have an effect estimate that would make the results of the meta-analysis insignificant, because the studies we included were limited and these studies reported quite different results. |
| Other compelling attributes of the data that may influence certainty | None |
| Overall strength of evidence | The strength of the evidence is inadequate, that is, residential noise might influent the risk of preeclampsia. Chance, bias, and confounding can be ruled out with reasonable confidence. Dose response analysis indicated that the risk of outcome did not increase with the increase of exposure levels. Moreover, because of the small number of included studies and the varying outcomes of these studies, we think that our conclusions may be influenced by the results of future studies. |
| **Residential noise to GDM** |  |
| Quality of body of evidence | Low |
| Direction of effect estimate | No significant relationship was observed between exposure to residential noise and preeclampsia. |
| Confidence in effect estimate | A new study would likely have an effect estimate that would make the results of the meta-analysis insignificant, because the studies we included were limited and these studies reported different results. |
| Other compelling attributes of the data that may influence certainty | None |
| Overall strength of evidence | The strength of the evidence is inadequate, that is, residential noise might influent the risk of GDM. Chance, bias, and confounding can be ruled out with reasonable confidence. Dose response analysis indicated that the risk of outcome did not increase with the increase of exposure levels. The available evidence includes the results of three high-quality studies, but because of the small number of included studies and the varying outcomes of these studies, we think that our conclusions may be influenced by the results of future studies. |

Abbreviations: GDM, gestational diabetes mellitus; HDP, hypertensive disorders of pregnancy.
